# The effect of grape interventions on cognitive and mental performance in healthy participants and those with mild cognitive impairment: a systematic review of randomized controlled trials

**DOI:** 10.1093/nutrit/nuab025

**Published:** 2021-05-26

**Authors:** Rachel Jayne Bird, Nigel Hoggard, Magaly Aceves-Martins

**Affiliations:** Rowett Institute, University of Aberdeen, Aberdeen, United Kingdom. M. Aceves-Martins is with the Health Services Research Unit, University of Aberdeen, Aberdeen, United Kingdom

**Keywords:** cognition, grape, memory, mood, Vitis

## Abstract

**Context:**

The prevalence of cognitive and mental health disorders are growing, and existing drug therapies do not treat the underlying cause. Grapes are a flavonoid-rich soft fruit and may therefore be beneficial to cognitive and mental health.

**Objective:**

To systematically review evidence from randomized controlled trials investigating the acute and chronic effects of grape interventions on measures of cognition and mood in healthy participants and those with mild cognitive impairment.

**Data Sources:**

MEDLINE, The Cochrane Library and EMBASE were searched.

**Data Extraction and Analysis:**

Eight studies met the inclusion criteria: one considered acute interventions, 6 assessed chronic effects, and one assessed acute and chronic effects of grapes. The chronic studies found improvements in some cognitive domains (eg, memory, motor skills, or executive function). Acute studies found no consistent effect on memory but saw improvements in reaction time.

**Conclusions:**

Differences in study design, dosages, and outcome tests hindered between-study comparison. Even so, the results across studies show that grapes can enhance some aspects of cognition, after both acute and chronic interventions.

**Systematic Review Registration:**

PROSPERO registration no. CRD42020193062.

## INTRODUCTION

The prevalence of mental and cognitive health disorders such as depression and dementia are growing worldwide, and existing drug therapies are unable to treat the underlying cause.^1^ Consequently, there is a need to discover interventions capable of preventing and slowing the development of these conditions. Lifestyle factors such as diet and exercise are integral to some of the most effective approaches,^2^ and they are not subject to the side effects and interactions that pharmacological treatments can have.

Specific aspects of diet, and in particular high intakes of fruit subgroups, including berries, have been shown to promote optimism and self-efficacy, as well as to reduce psychological distress and protect against depression.^3^ In addition, soft fruits have been shown to have positive effects on memory and cognition. For example, studies assessing mixed-berry drinks found working memory improved over a 5-week period,^4^ and sustained or improved cognitive function (eg, attention or memory) over 6 hours.^5^^,^^6^ The results of these studies indicate positive effects on memory and cognition after both acute and longer-term interventions and suggest that berries may have a preventative potential against cognitive decline.^4^

The positive effects of soft fruit are thought to be underpinned by their phytochemical, and in particular flavonoid content, and to involve a number of mechanisms, including neuroprotective properties, enhancing neuronal function, and stimulation of neurogenesis.^7^ The effects of flavonoid-rich food have been reported (in both clinical and observational studies) to improve measures of cognition and mood, particularly in relation to reducing cognitive decline in older adults,^8^^,^^9^ and evidence suggests flavonoid-rich soft fruits may be capable of delaying the progression of Alzheimer’s disease.^10^

Given the evidence for the positive effects of fruits in general, mixed berries, and flavonoid-rich foods on mental and cognitive health, assessment of the specific effects of individual soft fruits is needed. Grapes and blueberries are among the major fruit dietary sources of flavonoids, and they are high in 3 specific flavonoid subgroups; anthocyanins, flavan-3-ols, and flavonols.^10^ Several other soft fruits high in flavonoids, such as blackcurrants and blackberries, show promising effects in mental and cognitive health in humans and animals.^9^^,^^10^ However, the studies are limited in number compared with those that have been done on grapes and blueberries.

The large body of randomized controlled trials (RCTs) assessing the effects of blueberries on cognition and mood in humans has shown positive effects on cognition in both the short and longer term. A range of population groups have been studied, including children, young adults, healthy older adults, and older adults with mild cognitive impairment (MCI). Results suggest blueberry interventions may benefit cognitive performance in older people.^8^^,^^9^

There is also evidence for positive effects from grapes on cognitive function and mood, particularly in older adults with MCI.^1^ A critical review of epidemiological studies and RCTs assessing grapes and their derivatives, including wine, found positive effects in modulating the early stages of cognitive decline. Encouraging results were obtained in tests that measured reaction times, verbal skills, degree of orientation, learning, and memory.^10^ However, a systematic review of RCTs that have evaluated the effect of grape interventions on cognitive and mental health has, to date, not been undertaken. This study aimed to systematically review RCTs assessing the effect of acute and chronic grape dietary interventions on healthy subjects or on those with MCI.

## METHOD

A review protocol was registered in PROSPERO (registration number CRD42020193062).^11^ This review followed the Preferred Reporting Items for Systematic Reviews and Meta-Analyses (PRISMA) guidelines as a methodological template.^12^ The systematic review strategy was based on the PICO (population, intervention, comparator, outcomes) framework (Table 1).

**Table 1:** PICOS criteria for inclusion of studies

P (population)	Adults (>18 y) human participants who were healthy or with MCI (MCI being defined as persons with a degree of cognitive decline or memory loss noticed by themselves or their family members but not affecting their ability to carry out everyday tasks)
I (intervention)	Randomized controlled trials of grape intervention, including juice, freeze-dried powder, supplement, or extract
C (comparator)	Placebo
O (outcomes)	Any measurement of cognitive performance (eg, attention or memory); the secondary outcome included any measurement of mental health (eg, depression, mood, or anxiety)

Only published studies performed after 2010 were eligible for inclusion, and those in languages other than English were not considered. A scoping review was undertaken to identify the types of published studies in the field and to aid the protocol and search strategy. MEDLINE, the Cochrane Library, and EMBASE were systematically searched in June 2020 to identify published RCTs investigating the effects of grape intervention on cognition and mood. The search strategy included terms and Boolean connectors such as: (“grape$” OR “Vitis”) AND (“cognitive health” OR “mental health” OR “cognitive impairment” OR “cognitive decline” OR “mood” OR “depression” OR “memory”).

Eligible studies met the following criteria: RCTs including adult (>18 years) human participants who were healthy or had MCI (MCI being defined as persons with a degree of cognitive decline or memory loss noticed by themselves or their family members but not affecting their ability to carry out everyday tasks)^13^; used a grape intervention (including juice, freeze-dried powder, supplement, or extract) with placebo controls and assessed cognition and mood using appropriate tests. Observational studies, reviews, abstracts, conference papers, study protocols, studies including wine as a variation of grapes, participants with neurodegenerative diseases (eg, Alzheimer’s disease), and those that did not report applicable outcome measures were excluded. The primary outcomes included any measurement of cognitive performance (eg, attention or memory), and the secondary outcome included any measurement of mental health (eg, depression, mood, or anxiety).

The data extraction form was developed using the PICO framework and was standardized to ensure that relevant data was collected. Primary data extracted included study (eg, authors, publication year, study design), participants (eg, total number, mean age), intervention (eg, type of grape, formulation, dose), comparator, outcomes, and results. Each of the outcomes evaluated in the trials was categorized according to the cognition domains proposed by Harvey 2019.^14^ These domains include: Sensation (eg, visual, auditory, tactile, gustatory, or olfactory abilities), Perception (eg, object recognition, organizational strategies), Motor skills and construction (eg, motor abilities, including manual dexterity and motor speed), Memory (eg, working memory, episodic memory, procedural memory), Executive functioning (eg, reasoning, problem-solving), Processing speed (eg, coding and tracking), Language/Verbal skills (eg, reading and comprehension). If the studies evaluated mood, depression, or anxiety, this was also considered in the extraction and analysis.

The titles and abstracts of the studies were screened according to the eligibility criteria independently by one reviewer author (R.B.), and to establish consistency, a second reviewer (MA-M) revised and screened a 20% of the total references retrieved. For any articles for which it was unclear whether the eligibility criteria had been met, a full-text review was undertaken. The full texts were revised by one reviewer author (R.B.) with a 20% check by a second author (M.A.-M.). In case of any disagreement, the third author was consulted (N.H.). Acknowledging the wide heterogeneity variation of the tools for measuring the outcomes, quantitative synthesis (ie, meta-analysis) was not feasible. For this reason, the effectiveness reported in the primary publication was used in this report, and a narrative approach was taken for data synthesis.

Two authors (R.B. and M.A.-M.) independently assessed each study for bias using the Cochrane Collaboration’s tool for assessing the risk of bias in RCTs to determine the level of bias of the studies and across studies.^15^ This tool includes criteria for assessing sequence generation; allocation concealment; blinding of participants, personnel, and outcome assessors; incomplete outcome data; and selective outcome reporting. Study criteria were evaluated for risk of bias as low, unclear, or high. In case of any disagreement, the third author (N.H.) was consulted.

## RESULTS

**Figure 1:**
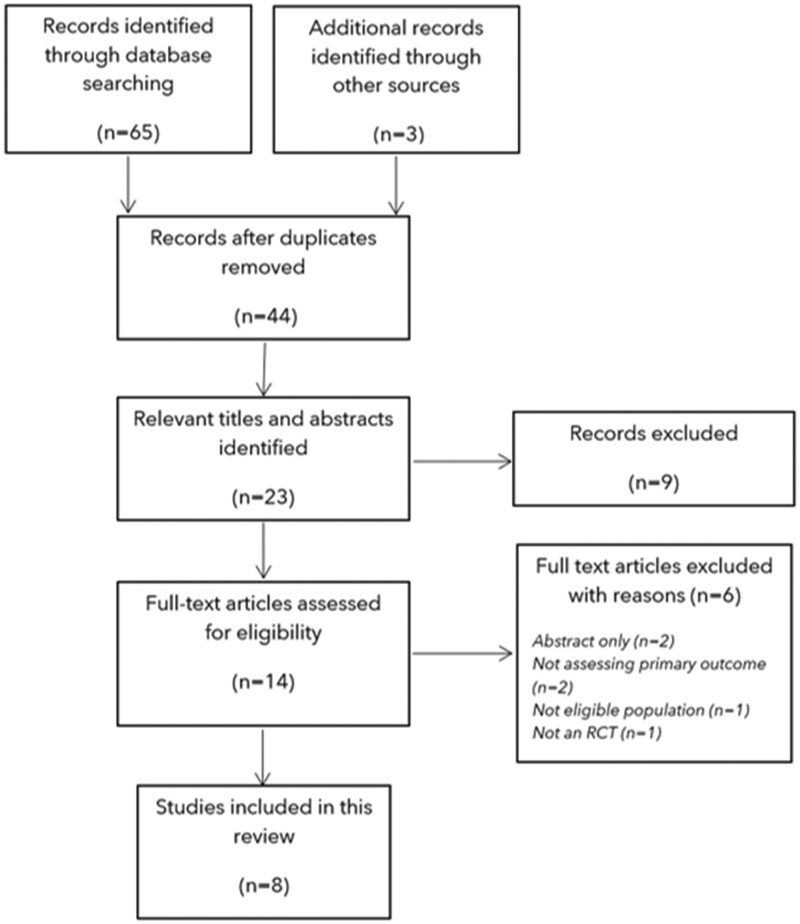
**PRISMA flow chart**.

In total, 65 papers were identified during our search, and 3 were identified from other sources. After de-duplication, 44 single references were identified. From these, 14 full-text articles were assessed for eligibility, leaving 8 RCTs^16–23^ for inclusion in this review (Fig. 1).

The total number of participants from the 8 included studies was 474, with sample sizes ranging from 10 to 215 participants (Table 2).^16–23^ All studies were RCTs, with 2 studies using a cross-over design,^16^^,^^19^ and the rest parallel design. Two studies were conducted on healthy young adults,^16^^,^^17^ 1 on healthy mothers,^18^ 2 on healthy older adults,^18^^,^^22^ and 3 investigated older adults with MCI.^20^^,^^21^^,^^23^ Three studies took place in England,^16^^,^^17^^,^^19^ 3 in the United States,^20^^,^^21^^,^^23^ 1 in Italy,^18^ and the bi-centric study took place in France and Canada.^22^ Overall, studies included a higher female proportion (ranging from 33% to 100%).

**Table 2:** Study characteristics summary

Reference	Study type	Population and gender	Mean age (SD); age range (y)	Country
Haskell-Ramsay et al (2017)^16^	Randomized, placebo-controlled, double-blind, counter-balanced crossover	20 healthy young adults (65% female)	21.2 (0.9); range 18–35	England
Bell et al (2020)^17^	Acute-on-chronic parallel groups, randomized, double-blind, placebo-controlled	60 healthy young adults (85% female)	20.9 (2.7); range 18–30	England
Calapai et al (2017)^18^	Randomized 2-group, parallel, placebo-controlled, double-blind	111 healthy older adults (52% female)	66.9 (5.2); range 56–75	Italy
Lamport et al (2016)^19^	Randomized, double-blind, placebo-controlled, crossover	25 healthy mothers (100% female)	43.2 (0.6); range 40–50	England
Krikorian et al (2010)^20^	Randomized, double-blind, placebo-controlled	12 older adults with mild cognitive impairment (33% female)	78.2 (5.0); range NR	USA
Krikorian et al (2012)^21^	Randomized, double-blind placebo-controlled	21 older adults with mild cognitive impairment (47% female)	76.9 (6.1); range: 68–90	USA
Bensalem et al (2019)^22^	Bi-centric, randomized, double-blind, placebo-controlled	215 healthy older adults (71.1% female)	64.66 (2.9); range 60–70	France and Canada
Lee et al (2017)^23^	Pilot. , randomized, double-blind placebo-controlled	Ten older adults with mild cognitive impairment (50% female)	72.2 (4.7); range NR	USA

*Abbreviations*: NR, Not Reported.

### Study intervention

The included studies used a range of grape interventions and control groups (Table 3).^16–23^ Four studies used *Vitis vinifera* (common grape),^17^^,^^18^^,^^22^^,^^23^ and 4 used *Vitis labrusca* (fox grape).^16^^,^^19–21^ One study used a polyphenol-rich extract from both grapes and blueberries,^22^ while the other studies solely used grapes as the intervention. Three studies used an inert placebo capsule containing maltodextrin,^17^^,^^18^^,^^22^ while 5 studies used a control that matched some components such as sugars and energy but which did not contain polyphenols.^16^^,^^19–21^ One study compared purple grape juice plus blackcurrant cordial with a control drink that contained white grape juice plus blackcurrant cordial^16^ and which had a significantly lower phenolic content compared with the intervention group. Two studies dosed the grape intervention based on the participant’s weight, increasing the dose with increasing weight.^20^^,^^21^

**Table 3:** Study interventions and comparators summary

References	Intervention	Placebo control	Study length
Haskell-Ramsay et al (2017)^16^	*Vitis labrusca*. Purple Concord grape juice. 230 mL drink once only The active treatment consisted of 200 mL Welch’s^®^ purple grape juice (based upon single-serving guidelines at the time of the study) plus 30 mL of Schweppes^®^ blackcurrant flavor cordial (containing 14 kcal and 3.1 g sugar per 100 mL). Schweppes™ blackcurrant flavor cordial was added at different volumes to the active treatment The nutritional content of drink: 140.2 kcal, 33.9 g of sugar, 138.3 mg/L anthocyanin content, 1504.5 μg/mL phenolic content	Sugar-matched intervention 230 mL drink containing 200 mL Welches white grape juice, 10 mL blackcurrant flavor, and 20 mL water The nutritional content of drink: 139.2 kcal, 33.9 g of sugar, 1.04 mg/L anthocyanin content, 135.1 μg/mL phenolic content	Acute: 20 min
Bell et al (2020)^17^	*Vitis vinifera*. 400 mg grape seed polyphenol extract delivered in capsule form (made of bovine gelatine), each matched for weight and containing 400 mg powder consumed once daily. The extract was a 400 mg mix of purified grape seed–derived polyphenolic extracts from *Vitis vinifera* (MegaNatural AZ; Polyphenolics Inc., Madera, CA, USA) containing catechin, epicatechin, proanthocyanins, and derivatives of catechin and epicatechin (epicatechin gallate) based on in-house analysis by Polyphenolics Inc. The nutritional content of the capsule: the exact calorie content of each was unknown.	Matched placebo capsule. A capsule containing 400 mg maltodextrin The nutritional content of the capsule|: the exact calorie content of each was unknown.	Acute: 4–6 h Chronic: 3 mo
Calapai et al (2017)^18^	*Vitis vinifera*. 250 mg oral supplement, once daily 250 mg of Cognigrape (Bionap srl, Italy). The extract consisted of proanthocyanins (>9%) and anthocyanins as malvidin-3-glucoside (4%–5%) The nutritional content of the capsule: not reported	Matched placebo capsule. Capsule containing maltodextrin The nutritional content of the capsule: not reported	Chronic: 3 mo
Lamport et al (2016)^19^	*Vitis labrusca*. 355 ml juice, once daily The nutritional content of the drink: 233 kcal, 59.5 g of carbohydrates, 54 g of sugars. 777 mg total polyphenolics as a gallic acid equivalent/355-mL daily serving (167 mg anthocyanins asmalvidin equivalent, and 334 mg proanthocyanins as catechin equivalent). Vitamin C was not present in the supplement.	Energy, appearance, taste, volume, carbohydrate content, and all sugars matched intervention. The nutritional content of the drink: 233 kcal, 59.5 g of carbohydrates, 54 g of sugars. No vitamin C or polyphenols present	Chronic: 3 mo
Krikorian et al (2010)^20^	*Vitis labrusca*. juice 3 times a day A drink based on 100% Concord grape juice provided by Welch Foods, Inc. (Concord, MA, USA). The nutritional content of the drink: drink had 0.7 kcal/mL dosed on body weight (daily consumption between 6 and 9 mL/kg). No further details of the drink were provided.	The drink contained no juice or natural polyphenol. However, it was formulated to look and taste like grape juice and to have the same carbohydrate composition and energy. The nutritional content of the drink: the drink had 0.7 kcal/mL dosed on body weight (daily consumption between 6 and 9 mL/kg).	Chronic: 3 mo
Krikorian et al (2012)^21^	*Vitis labrusca*. Dosed on body weight, juice 3 times a day A drink based on 100% Concord grape juice derived by hot press and pasteurized with no added ingredient provided by Welch Foods, Inc. (Concord, MA, USA) The nutritional content of the drink: 425 mg/L of anthocyanin content. 46% anthocyanins, 29% tartaric acid esters of hydroxycinnamates, and 10% procyanidins. No further information reported	Drink matching the grape juice concerning color, taste, total calories, and sugar profile, with no juice or polyphenolic compounds The nutritional content of the drink: no further information reported	Chronic: 4 mo
Bensalem et al (2019)^22^	*Vitis vinifera* and *Vaccinium angustifolium* Aiton. 300 mg capsule twice daily (1 capsule at least 1 h after breakfast and 1 capsule at least 1 h after dinner) Nutritional content of the capsule: 300 mg of extract, including low-molecular weight polyphenols: 42.8 ± 2.8% of total flavonoids (flavan-3-ols, flavanols, and anthocyanins) including 22.9 ± 1.6% of flavan-3-ols monomers and 19.1 ± 7.3% of oligomers, 0.6 ± 0.2% of flavanols (quercetin and glycosylated derivatives), 0.13 ± 0.04% of anthocyanins (especially malvidin 3-glucoside), 1.8 ± 0.6% of phenolic acids (such as chlorogenic, gallic, and ferulic acids); and 0.04 ± 0.01% of stilbenes (resveratrol). Two capsules provided 258 ± 17 mg of flavonoids.	A matched placebo capsule The nutritional content of the capsule: 300 mg of pure maltodextrin (ref Maltrin^®^ M100), providing no polyphenol	Chronic: 6 mo
Lee et al (2017)^23^	*Vitis vinifera*. Freeze-dried powder twice daily Active grape formulation (the freeze-dried grape powder made of commercially grown fresh red, green, and blue-black California grapes 36 g p.o. bid reconstituted in 227 mL of water) The nutritional content of the powder: total polyphenol content is 495 mg/100 g. Packet composed of 36 g of either formulation twice/day (total of 72 g/day, equivalent to 3 standard servings of fresh grapes per day). 458.9 mg/kg of anthocyanin content. No other information provided.	A matched placebo powder. Same content of fructose and glucose matched intervention but free from polyphenols The nutritional content of the powder: no information provided	Chronic: 6 mo

The duration ranged from acute intervention trials measuring outcomes after 20 minutes^16^ and 4 and 6 hours post consumption^17^ to chronic intervention trials of up to 6 months.^22^^,^^23^ One study assessed the effects of both acute and chronic grape interventions,^17^ 3 studies assessed the effects of chronic grape interventions over 3 months,^18–20^ 1 study length was 4 months,^21^ and 2 studies lasted 6 months.^22^^,^^23^ Four studies documented a range of anthocyanin contents for the grape intervention: the values reported were 138.3 mg/L,^16^ 167 mg,^19^ 425 mg/L^21^ 458.9 mg/kg.^23^ Flavanol content was reported in one trial, which included 88.23 mg/kg,^23^ but flavan-3-ols were not reported in any of the studies. Two studies provided values for what their intervention would be equivalent to: this was ∼200 g of fresh grapes^22^ and 3 standard servings of fresh grapes daily (Table 3).^16–23^

### Outcomes

A wide range of cognitive and mental health outcomes were used across the included studies (Table 4).^16–23^ According to the categorization of cognitive domains used (Harvey 2019),^14^ all the studies included measurements of memory as a primary outcome (eg, verbal, nonverbal, visuospatial, episodic, working, immediate, delayed, and self-reported memory). Furthermore, 4/8 measured “Language or Verbal skills,”^18^^,^^20^^,^^21^^,^^23^ 4/8 measured “Perception,”^16–18^^,^^22^ 3/8 measured “Executive functioning,”^17^^,^^19^^,^^23^ 2/8 measured “Motor skills and construction,”^17^^,^^19^ and none measured sensations. Seven of the included trials also included measures of mood, depression, or anxiety.^16–21^^,^^23^ All studies used a battery of tests, and the number ranged from 3^20^^,^^21^ to 25^23^ tests to assess outcome measures. The full list of tools and tests performed can be seen in Table 4.^16–23^

**Table 4:** Study outcomes and results summary

References	Outcomes measured	Cognitive domain						Mood, depression, or anxiety	Main results
		Sensation	Perception	Motor skills and construction	Memory	Executive functioning	Language or verbal skills		
Haskell-Ramsay et al (2017)^16^	Memory (immediate and delayed word recall, numeric working memory, word recognition, picture recognition) Attention (simple reaction time, choice reaction time, digit vigilance) Subjective mood (calm, content, alert)	×	✓	×	✓	×	×	✓	Acute results (20 min): improved perception (reaction time on a composite attention measure) (*P* = 0.047) No effect on memory outcomes Improved mood (increased calm ratings) (*P* = 0.046) No significant correlations between change in mood ratings and composite cognitive scores were observed.
Bell et al (2020)^17^	Language or verbal skills (auditory verbal learning test, serial subtraction 3 s and 7 s, the Modified Attention Network Test) Motor skills (simple and complex finger tapping and the switching task) Memory (retention of words over different time-lapses) Executive function (switching task) Subjective mood Mental fatigue	×	✓	✓	✓	✓	×	✓	Overall, outcomes showed no significant effects of treatment for acute and chronic analysis Acute analysis (4–6 h): Improved executive function (faster responses for switching tasks) (*P* < 0.01) relative to placebo at 2 h and 4 h Improved attention (faster scores in Modified Attention Network Test) (*P* < 0.001), but this result was also seen in the placebo group Chronic analysis (3 mo): Improved motor skills (finger tapping) (*P* < 0.05) whereby performance decreased between weeks 6 and 12 for the placebo (*P* < 0.05) Mood overall was not improved
Calapai et al (2017)^18^	Memory, attention, and language (Mini-Mental State Examination) Depression (Beck Depression Inventory) Anxiety and Mood (Hamilton Anxiety Rating Scale) Repeatable Battery for the Assessment of Neuropsychological Status	×	✓	×	✓	×	✓	✓	Chronic analysis (3 mo): Improvements relative to placebo and baseline data Improvement in memory, attention, and language (Mini-Mental State Examination scores improvement, *P* < 0.0001) Improved Repeatable Battery for the Assessment of Neuropsychological Status (*P* < 0.001), attention (*P* < 0.001), language (*P* < 0.05), immediate and delayed memory (both *P* < 0.0001) Improvement in depression and anxiety. Scores of Beck Depression Inventory (*P* < 0.0001) and Hamilton Anxiety Rating Scale (*P* < 0.05) reduced
Lamport et al (2016)^19^	Language or verbal skills and memory (visual verbal learning test, immediate recall–verbal memory, visual spatial learning test immediate recall–nonverbal spatial memory, Rey Auditory Verbal Learning Test) Executive function (rapid visual information processing, Tower of Hanoi) Motor skills and construction (psychomotor skill, grooved pegboard, driving performance.) Subjective mood, stress, anxiety (100-mm visual analog scales with questions, Perceived Stress Scale, short State–Trait Anxiety Inventory)	×	×	✓	✓	✓	×	✓	Chronic analysis (3 mo): Improvements relative to placebo and to baseline data Improvement in immediate spatial memory and driving performance relative to placebo (*P* < 0.05). Visual spatial learning test immediate recall showed a significant main effect of condition recall higher after the intervention (no *P*-value presented). Improvement in executive function (completion time was faster after the intervention) (*P* < 0.01). Driving performance was more accurate and better scores were recorded for the intervention group (*P* < 0.05). Alertness and concentration were significantly higher for participants in the intervention group (*P* < 0.05). There were no improvements in subjective mood, stress, or anxiety.
Krikorian et al (2010)^20^	Memory and language or verbal skills (verbal learning, retention, nonverbal memory by using The California Verbal Learning Test) Mood and depressive symptoms (Geriatric Depression Scale)	×	×	×	✓	×	✓	✓	Chronic analysis (3 mo): Improvements relative to placebo and to baseline data Improved verbal learning. Significant effect (*P* = 0.04) for item acquisition across learning trials on the California Verbal Learning Test No significant effect on depressive symptoms, mood, or enhancement of verbal and spatial recall memory
Krikorian et al (2012)^21^	Memory and language or verbal skills (Montreal Cognitive Assessment and Rey Auditory Verbal Learning Test, the California Verbal Learning Test-II) Mood and depressive symptoms (Geriatric Depression Scale) Brain activation during working memory tests, mood	×	×	×	✓	×	✓	✓	Chronic analysis (4 mo): Enhanced neurocognitive functions (significant effect for right middle frontal cortex, *P* = 0.05, and marginally significant effect for right superior parietal cortex *P* = 0.07), indicating greater activation in the grape group compared with the placebo group Reduced semantic interference on memory tasks, but no other effect on memory and language or verbal skills No effect on mood
Bensalem et al (2019)^22^	Memory (visuospatial learning and episodic memory test, episodic verbal recall memory using the VRM free recall test, working memory, verbal recall memory)	×	✓	×	✓	×	×	×	Chronic analysis (6 mo): improvements relative to placebo and to baseline data No significant difference was observed between intervention and placebo groups in verbal recall and working memory. However, there was improved verbal episodic and recognition memory performance (*P* = 0.006). There was improved cognitive and memory performance compared with baseline scores, but not compared with the placebo group.
Lee et al (2017)^23^	Memory (Hopkins Verbal Learning Test-Revised, Benton Visual Retention Test, Rey–Osterreith Complex Figure Test delayed, visuospatial–Rey–Osterreith Complex figure test copy, attention and working memory WAIS-III Letter–Number Sequencing) Language or verbal skills (Boston Naming Test, Letter Fluency FAC, Category Fluency, Estimated Verbal IQ Wechsler Test of Adult Reading) Executive function (Stroop Interference, Trail Making Test–Part B, Wisconsin Card Sorting Test-64, Speed of information Processing, WAIS-III Digital Symbol, WAIS-III Symbol speed) Depression and anxiety (Hamilton Depression Rating Scale and Hamilton Anxiety Rating Scale) Neuroimaging tests	×	×	×	97	✓	✓	✓	Chronic analysis (6 mo): Analysis relative to baseline data. No differences between the groups presented in the paper There were no significant changes among any of the measured outcomes (*P* > 0.05) Decline in the metabolism of the right posterior cingulate cortex (*P* = 0.01) and left superior posterolateral temporal cortex (*P* = 0.04) in the placebo group. These regions are known to be significantly affected in the early stages of Alzheimer’s disease, and the decline was seen in the placebo group, but not the active grape formulation group. This would suggest a beneficial effect on the intervention group.

### Cognitive domain health outcomes

####  

*Memory.* A variety of memory tests were used across the studies. Of the 8 studies, 2 reported no effects of the intervention on memory outcomes,^16^^,^^17^ 4 reported a significant beneficial effect over memory,^18–20^^,^^23^ and the other 2 studies reported different results across the different types of memory tests.^21^^,^^22^ For example, Krikorian et al^21^ found enhanced neurocognitive functions, reduced semantic interference on memory tasks, but no other effect on memory outcomes after 4 months of grape juice intervention.

####  

*Language or verbal skills.* A variety of language and verbal skills tests were used across the studies. Some of the tools used to measure these were composite tests, meaning they included more outcomes measurements. Of the 4 studies that measured this domain, 1^18^ reported improvements in acute and chronic analysis, 1^20^ reported improvements relative to placebo and to baseline data in chronic analysis (after 3 mo), and 2 did not report any effect in this domain.^21^^,^^23^

####  

*Perception.* A variety of perception tests were used across the studies. Of the 4 studies measuring this domain, 2 studies reported improvements in both the acute and chronic intervention analysis, but in the other 2 studies the results regarding perception were unclear or biased. For instance, Bell et al^17^ reported improved attention (faster scores in Modified Attention Network Test) (*P* < 0.001), but improvement was also seen in the placebo group. Calapai et al^18^ found improvements in the Repeatable Battery for the Assessment of Neuropsychological status (RBANs) test, which is validated for elderly subjects and used for dementia diagnostic purposes. Furthermore, improvements in attention (*P* < 0.001) and language (*P* < 0.05) in healthy older adults after grape intervention over 3 months (compared with placebo) were also reported.

####  

*Executive functioning.* Three studies measured this domain. Two^17^^,^^19^ reported improvements in executive function, and one did not report the results for this domain.^23^ Improvements reported in this domain included faster responses for switching tasks (*P* < 0.01)^17^ (relative to those of the placebo group) at 2 hours and 4 hours, and faster completion times on tasks after the intervention (*P* < 0.01).^19^

####  

*Motor skills.* Two studies measured this domain. One study^17^ reported improved motor skills, measured as finger tapping (*P* < 0.05), with the grape intervention, whereas performance decreased between weeks 6 and 12 for the placebo (*P* < 0.05). The second study,^19^ reported that driving performance was more accurate and better scores were recorded for the intervention group (*P* < 0.05).

### Mental health outcomes

Seven studies with a combined sample size of 259 participants assessed the secondary outcome: mood, depression, or anxiety.^16–21^^,^^23^ Of these, 5 studies demonstrated no appreciable effect on mood symptoms,^17^^,^^19–21^^,^^23^ and 1 study found a benefit only in measures of calmness after acute exposure (20 min) to grapes.^16^ The only study to report significant improvements in mood used the Mini-Mental State Examination, Beck Depression Inventory, and Hamilton Anxiety Rating Scale scores, which indicated improvements in mood, and reduction in depression and anxiety symptoms after grape supplement compared with placebo, in healthy older adults over a 3-month study period.^18^

### Risk of bias

The risk of bias assessment was performed on all studies (Table 5).^16–23^ The overall risk was deemed low for 2 trials^17^^,^^18^ and unclear for 6 trials.^16^^,^^19–23^ One study was observed to have an unclear risk for “other bias” due to it using white grape juice as the placebo for purple grape juice intervention and a 6- to 7-day washout period between cross-over of arms.^16^ It was deemed to have unclear risk of bias due to the potential for the placebo grape and the short washout period to influence the study results. Five of the studies were deemed to have an unclear risk of bias with respect to allocation concealment, because the method used to conceal the allocation sequence was not described.^16^^,^^19^^,^^21–23^ All trials detailed blinding of participants and personnel.

**Table 5:** Risk of bias summary

References	Random sequence generation	Allocation concealment	Blinding of participants and personnel	Blinding of outcome assessment	Incomplete outcome data	Selective reporting	Other
Haskell-Ramsay et al (2017)^16^	Unclear	Unclear	Low	Low	Unclear	Low	Unclear
Bell et al (2020)^17^	Low	Low	Low	Low	Low	Low	Low
Calapai et al (2017)^18^	Low	Low	Low	Low	Low	Low	Low
Lamport et al (2016)^19^	Low	Low	Low	Low	Unclear	Low	Unclear
Krikorian et al (2010)^20^	Unclear	Unclear	Low	Low	Low	Low	Unclear
Krikorian et al (2012)^21^	Unclear	Unclear	Low	Low	Low	Low	Low
Bensalem et al (2019)^22^	Low	Unclear	Low	Low	Unclear	Low	Low
Lee et al (2017)^23^	Unclear	Unclear	Low	Low	Low	Low	Unclear

## DISCUSSION

This study aimed to systematically review RCTs assessing the effect of acute and chronic dietary grape interventions on healthy subjects or those with MCI. Our searches identified 8 RCTs that met our inclusion criteria. As expected, outcomes measurements varied widely among the included studies. However, the results of the included trials suggested that several formulations of grape interventions did enhance some aspects of cognition. Some of the improvements reported included enhanced speed in the attention tasks or neurocognitive functions, improvement in immediate spatial memory, improvement in driving performance, and reduced semantic interference in memory tasks. However, the evidence showed improvement in some, but not all, of the cognitive domains considered. For instance, motor skills and executive function seemed to be improved across the studies. However, domains like memory or perception showed different results across the studies, making the evidence inconclusive. Furthermore, some results indicated no change when using other assessment tools or outcome measures.

Similar to this review, findings from a review of 15 human RCTs found a positive association between the consumption of various flavonoids (ie, soy isoflavones, cocoa, and *Ginkgo* *biloba*) and cognitive function.^7^ However, the authors of that review also found that study comparisons were difficult due to the lack of consistency and considerable heterogeneity in outcome measurements and study design. In addition to that, 27 different cognitive outcome domains were assessed across the studies in the current review. A range of assessment tools and tasks are used in studies investigating the effect of dietary interventions on cognition and mental health outcomes, because there is no one set gold-standard test for cognitive and mental health outcomes worldwide. The overall quality was unclear for most of the trials in this review (6/8), and no high-quality research was identified. Studies mainly missed reporting the methods for allocation concealment or random sequence generation. Also, some of the studies had incomplete outcome data, there were different types of study designs (eg, parallel and cross-over designs), and the various tools used to assess outcomes are diverse, making it difficult to estimate the effect size of interventions in this field (which could be done in a meta-analysis).

Findings from the chronic intervention studies (3–6 mo) in this review indicated that cognitive benefits may be found in the form of improved verbal learning in adults with MCI,^20^ improved spatial memory in healthy mothers,^19^ and improved attention, language, and memory in healthy older adults.^18^ Acute intervention studies (measuring outcomes at between 20 min and 6 h post-consumption) reported no consistent effect on memory, but saw improvements in reaction time and responses in healthy young adults.^16^^,^^17^ Similar to these findings, the results from a critical review of epidemiological studies and RCTs of grape interventions in humans also found positive effects on verbal skills and learning, memory, reaction times, and degree of orientation.^11^

Positive effects on cognition were also reported in healthy older adults,^18^ healthy mothers,^19^ and adults with MCI,^20^^,^^21^ but (conversely) no beneficial cognitive effects were also reported in healthy adults^17^ and older adults with MCI.^21^ Inconsistencies in outcome measures, type of grapes, doses, and flavonoid content may account for the discrepancies in results between trials. However, overall, the cognitive results suggest that longer-term use of grapes is more likely to have a positive effect on memory than acute exposure.

There is less evidence suggesting positive benefits of grape intervention on mood. Only one study found improvements in depression and anxiety symptoms after 3 months of exposure to grapes,^18^ while most studies found no difference in mood. A reason for the difference in results between domains may be due to flavonoids providing significantly more benefit to specific cognitive domains such as memory, rather than mood.

The findings from the current systematic review are similar to those from reviews of blueberry interventions that found benefits in certain age groups, in some acute interventions and some chronic interventions, and in some cognitive (eg, short- and long-term memory and spatial memory) and mental health assessments, but not others (eg, mood). The blueberry reviews gave the authors an incomplete picture, but they concluded that blueberry polyphenols can improve some aspects of cognition across certain areas.^8^^,^^9^ Blueberries and grapes both contain the flavonoid subgroup anthocyanins in relatively high amounts: blueberries approximately 163.3 mg/100 g; grapes approximately 48.1 mg/100 g,^24^ which may explain why similar effects are seen. However, flavonoid concentrations were not reported in all the trials looked at in the current review. In addition, the doses of the grape interventions relative to human portion sizes were only reported in 2 out of the 8 trials. This limited the scope to compare outcomes and attribute the benefits of grapes to their specific flavonoid type, quantity, or dose of intervention. None of the included studies quantified the polyphenol content of grapes, including the anthocyanins, flavonols, and flavon-3-ols. As a guide, the studies examined in the critical review by Restani et al^11^ suggest a 200–500 mL/day consumption of grape juice is correlated with positive effects on cognitive performance. The most effective length of intervention for cognitive and mental health performance has not been established, and hence recommendations about time frames cannot be made from this review data.

The studies in this review included different population sizes and varying demographics, such as age and proportion of females recruited. Generally, more females than males were studied, and the sample sizes were relatively small, with 5 of the studies’ sample sizes being 25 or less, making comparisons between trials, and reliable conclusions, more challenging. In future studies, sample sizes should be calculated based on expected effect size; otherwise, the power of an intervention to detect a difference between treatment and placebo is uncertain.^7^

This systematic review has some limitations. First, there was heterogeneity in the included studies in sample size, dose, measurement tools, etc., which caused difficulties in the appropriate synthesis of the effectiveness of grape consumption. Second, most of the included trials had an unclear risk of bias assessment. However, a specific strength of this systematic review is that, to our knowledge, this is the first systematic review that has only included RCTs (considered the gold standard for evaluating healthcare outcomes).

Based on the promising results for grapes’ positive effects on cognitive function, particularly after chronic use, further research in this area is recommended, predominantly investigating the cognitive outcomes of memory, reaction time, and learning. Future research should be done in larger population sizes. Such studies need to be adequately powered, and to define the polyphenol content of the grape interventions and doses relative to portion sizes so that the optimal whole-fruit grape dose can be determined to inform human dietary guidelines. Further research into whether grape interventions have consistent positive effects on cognitive and/or mental performance in healthy participants and those with MCI is needed to be able to make recommendations about using dietary or supplementary grapes to prevent cognitive and mental health disorders and/or slow down the progression of established MCI.

## CONCLUSION

Evidence suggests that grapes might have a positive effect on cognitive health after both acute and chronic interventions. In particular, some aspects of the memory domain as well as motor skills and executive functions may derive benefit. However, with the current level of evidence, no conclusion can be reached as yet about the positive effects of grapes on mood, or the age group or health status of the population that may benefit, either from acute or chronic exposure. High-quality research is required (including longer-term RCTs) to measure more accurately the effects of grapes on cognitive and mental health.
